# Replenishable prevascularized cell encapsulation devices increase graft survival and function in the subcutaneous space

**DOI:** 10.1002/btm2.10520

**Published:** 2023-05-19

**Authors:** Gauree S. Chendke, Bhushan N. Kharbikar, Sudipta Ashe, Gaetano Faleo, Julie B. Sneddon, Qizhi Tang, Matthias Hebrok, Tejal A. Desai

**Affiliations:** ^1^ UC Berkeley ‐ UCSF Graduate Program in Bioengineering San Francisco California USA; ^2^ Department of Bioengineering and Therapeutic Sciences University of California San Francisco San Francisco California USA; ^3^ Diabetes Center, University of California, San Francisco San Francisco California USA; ^4^ Department of Surgery UCSF Gladstone Institute of Genome Immunology San Francisco California USA; ^5^ Department of Cell and Tissue Biology University of California, San Francisco San Francisco California USA; ^6^ Department of Anatomy University of California, San Francisco San Francisco California USA; ^7^ Eli and Edythe Broad Center of Regeneration Medicine and Stem Cell Research San Francisco California USA; ^8^ Center for Organoid Systems, Technical University Munich Garching Germany; ^9^ Institute for Diabetes Organoid Technology, Helmholtz Munich, Helmholtz Diabetes Center Neuherberg Germany; ^10^ School of Engineering, Brown University Providence Rhode Island USA

**Keywords:** beta cell replacement therapy, cell encapsulation device, human stem cells, prevascularization, transplantation in subcutaneous space, type 1 diabetes

## Abstract

Beta cell replacement therapy (BCRT) for patients with type 1 diabetes (T1D) improves blood glucose regulation by replenishing the endogenous beta cells destroyed by autoimmune attack. Several limitations, including immune isolation, prevent this therapy from reaching its full potential. Cell encapsulation devices used for BCRT provide a protective physical barrier for insulin‐producing beta cells, thereby protecting transplanted cells from immune attack. However, poor device engraftment posttransplantation leads to nutrient deprivation and hypoxia, causing metabolic strain on transplanted beta cells. Prevascularization of encapsulation devices at the transplantation site can help establish a host vascular network around the implant, increasing solute transport to the encapsulated cells. Here, we present a replenishable prevascularized implantation methodology (RPVIM) that allows for the vascular integration of replenishable encapsulation devices in the subcutaneous space. Empty encapsulation devices were vascularized for 14 days, after which insulin‐producing cells were inserted without disrupting the surrounding vasculature. The RPVIM devices were compared with nonprevascularized devices (Standard Implantation Methodology [SIM]) and previously established prevascularized devices (Standard Prevascularization Implantation Methodology [SPVIM]). Results show that over 75% of RPVIM devices containing stem cell‐derived insulin‐producing beta cell clusters showed a signal after 28 days of implantation in subcutaneous space. Notably, not only was the percent of RPVIM devices showing signal significantly greater than SIM and SPVIM devices, but the intraperitoneal glucose tolerance tests and histological analyses showed that encapsulated stem‐cell derived insulin‐producing beta cell clusters retained their function in the RPVIM devices, which is crucial for the successful management of T1D.

## 1 INTRODUCTION

Type 1 diabetes (T1D) is an autoimmune disease in which the immune system attacks insulin‐producing beta cells in the pancreas. Loss of beta cells results in an inability to produce and secrete adequate levels of insulin, especially in response to changes in peripheral glucose levels, which can lead to hyperglycemia and several long‐term complications.^1^, ^2^, ^3^ People with T1D must frequently monitor blood glucose levels and require exogenous insulin delivered through pumps or injections. While this strategy helps manage T1D, under and overdosing on exogenous insulin may lead to hyperglycemia and hypoglycemia, respectively, leading to long‐term morbidity. A longer‐term solution for treating T1D is islet transplantation, which replaces the destroyed cells with functional beta cells.^1^, ^2^, ^4^, ^5^, ^6^ This therapy can recapitulate endogenous beta cells' intricate glucose sensing and insulin‐releasing capabilities while eliminating the burdens of patient compliance and dependence on exogenous insulin. A drawback to this therapy is that patients are prescribed long‐term immunosuppressive drugs to prevent immune rejection of transplanted cells, which can be fatal. Cell encapsulation using macroencapsulation devices have shown to be a promising approach to address this challenge.^1^, ^2^, ^7^, ^8^, ^9^, ^10^, ^11^, ^12^, ^13^ Encapsulation devices offer a physical barrier to protect transplanted beta cells from immune attack; however, this barrier can limit the diffusion of oxygen and nutrients, causing ischemic stress detrimental to graft survival. This ischemic stress varies based on the transplantation site of the device.^1^, ^2^, ^4^, ^7^, ^8^, ^9^, ^10^, ^14^, ^15^

An ideal transplantation site for cell encapsulation devices includes (1) a dense vascular network that allows for insulin and glucose exchange, along with high oxygen and nutrient supply to the graft; (2) a hospitable microenvironment that prevents initial loss of cells posttransplant; (3) a minimally invasive procedure for implanting, monitoring, and retrieving the graft. Transplantation in the subcutaneous space allows for minimally invasive implantation and retrieval.^5^, ^11^, ^12^, ^16^, ^17^, ^18^, ^19^, ^20^, ^21^, ^22^, ^23^ However, a significant challenge that remains unaddressed in subcutaneous cell transplantation is the loss of beta cell viability that occurs shortly after transplantation. During surgical implantation, the inherently low vasculature in the subcutaneous space is further destroyed, leading to an even lower supply of oxygen and nutrients at the transplantation site. This is particularly detrimental to highly metabolic beta cells.^1^, ^2^, ^7^, ^16^, ^17^, ^22^, ^24^, ^25^

Several studies have shown that subcutaneous transplantation sites can be modified to promote neovascularization posttransplantation. These methods involve the use of biologics such as growth factors (beta‐fibroblast growth factor [FGF], vascular endothelial growth factor),^26^, ^27^, ^28^ chemical modifications of the encapsulation material,^29^ anti‐inflammatory drugs,^14^, ^30^, ^31^ co‐delivery of mesenchymal stem cells,^32^, ^33^, ^34^ and the use of oxygen generators,^10^, ^35^, ^36^, ^37^ among others. While these strategies may improve blood vessel formation, these methods require at least 10 days to create a dense vascular network that is well integrated with the host vasculature after cells have already been implanted in the subcutaneous space. Due to this limitation, these methods cannot rescue most of the graft loss that occurs within the initial days of implantation.^7^, ^11^, ^12^, ^15^, ^21^, ^38^

Another promising strategy to improve islet vascularization is the prevascularization of encapsulation devices at the transplantation site. In this approach, a non‐vascularized device is implanted before transplanting beta cells in the vascularized site. The advantage of this approach is that the host vasculature is well incorporated at the transplantation site, improving access to oxygen and nutrient supplies.^7^, ^11^, ^20^, ^21^, ^32^, ^39^, ^40^ However, a drawback of this strategy is that the vascularized device is typically removed prior to beta cell transplantation. Device removal can rupture some newly formed vascular networks and create a suboptimal microenvironment for subsequent islet transplantation. Several groups have sought to overcome this drawback; however, these approaches involve in vitro prevascularized devices or more complex encapsulation devices that require different membranes, drugs, and surface modification to promote in vivo vascular growth.^7^, ^41^, ^42^, ^43^ To our knowledge, there are currently no strategies for encapsulation devices that create a prevascularized transplantation site that allows for the direct insertion of cells without disrupting the surrounding vasculature.

Here we report a prevascularization strategy using a replenishable encapsulation device that prevents the initial loss of cell viability and function of stem cell‐derived insulin‐producing beta cell clusters in the subcutaneous space. Our method was designed to prepare the transplantation site such that a functional vascular network surrounds the encapsulation device prior to the transplantation of cells. This approach was developed using thin‐film polycaprolactone (PCL) cell encapsulation devices that have been previously shown to maintain the viability and function of insulin‐producing cells in the liver lobe for at least 6 months.^44^ We have also demonstrated that such devices support the viability of insulin‐producing cells in the subcutaneous space by incorporating nutrient depots.^24^ Devices were implanted subcutaneously for 2 weeks, after which, they were loaded with stem cell‐derived insulin‐producing beta cell clusters without disrupting the integrity of the surrounding vascular network. After 28 days, more than 80% of prevascularized replenishable devices showed signal, leading to a measurable C‐peptide secretion in response to a glucose challenge.

## 2 RESULTS

### 2.1 Design of subcutaneous thin film PCL device and implantation technique

**FIGURE 1:**
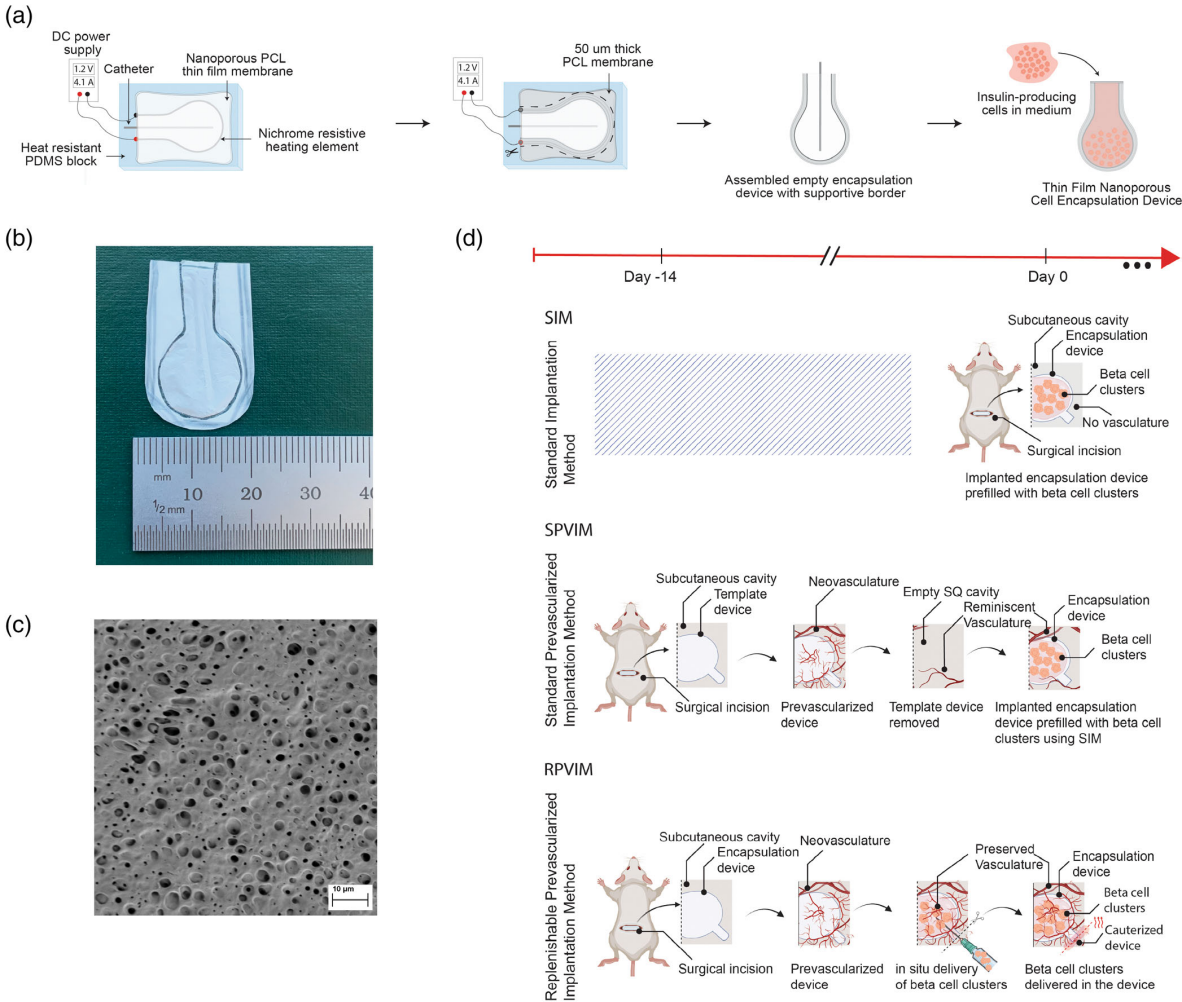
Schematic of cell encapsulation device and prevascularization methodology. (a) Illustration of the fabrication of thin film polycaprolactone (PCL) encapsulation devices assembled using a resistive heating method. (b) Image of the 2 cm wide encapsulation device showing the long neck that allows for easy insertion of cells and thicker surrounding membrane that provides mechanical support. (c) Cross‐sectional SEM image of nanoporous PCL membrane used to fabricate encapsulation devices. The inner pores of the membranes are ~200 nm in size, and the membrane thickness is ~10 μm. (d) Implantation strategies for comparing standard implantation, standard prevascularization implantation, and replenishable prevascularization implantation methods. RPVIM, Refillable Prevascularized Implantation Method; SIM, Standard Implantation Method; SPVIM, Standard Prevascularization Method.

To create a refillable device, we modified the fabrication of thin film devices described in previous work.^24^, ^45^, ^46^ We fabricated a small device that was 2 cm in length and width to permit the insertion of the device in the subcutaneous space with minimal stress to the animal. In brief, the new design included a 1 cm long and 0.6 cm wide neck with an enclosed catheter that allows for a facile opening of the device and the insertion of cells (Figure 1). The long neck was crucial in opening the device and inserting cells without removing the device from the transplant site and disrupting the surrounding vasculature. After inserting the cells, a cauterizer was used to seal the device opening through resistive heating. The circular region, where the cells reside, has a diameter of 1.6 cm and maximum volume capacity of 160 μL. To provide mechanical support, we also incorporated a 100 μm thick PCL border around the device, which keeps it sturdy and prevents it from folding over in vivo. Additionally, transplantation in the subcutaneous space allowed for multiple surgeries where no adverse side effects were observed (based on whole animal and gross site observation). This may not be possible with other implantation sites, where repeated administration may lead to greater adverse events.^16^, ^17^, ^19^

Once encapsulation devices were assembled, we next determined the ability of devices to maintain cellular viability and function, with and without prevascularization and/or device removal. Devices were either loaded with cells or immediately transplanted into an unmodified subcutaneous space (Standard Implantation Method [SIM]), or were first implanted as an empty device to establish vasculature before loading with cells (Refillable Prevascularized Implantation Method, RPVIM). For the prevascularized devices, an empty device was allowed to vascularize for 14 days. After that, stem cell‐derived insulin‐producing beta cell clusters were inserted, and devices were sealed without disrupting the newly formed vasculature around the device. To demonstrate that maintaining an intact vascular network in RPVIM is critical for graft survival, we also compared with a previously reported approach where prevascularized empty devices were explanted and replaced with a SIM device (Standard Prevascularization Method [SPVIM]). We hypothesized that disruption of the prevascularized zone around the device would limit its ability to preserve graft survival in the subcutaneous space.

### 2.2 Vasculature formation after transplantation of encapsulation device in subcutaneous space

**FIGURE 2:**
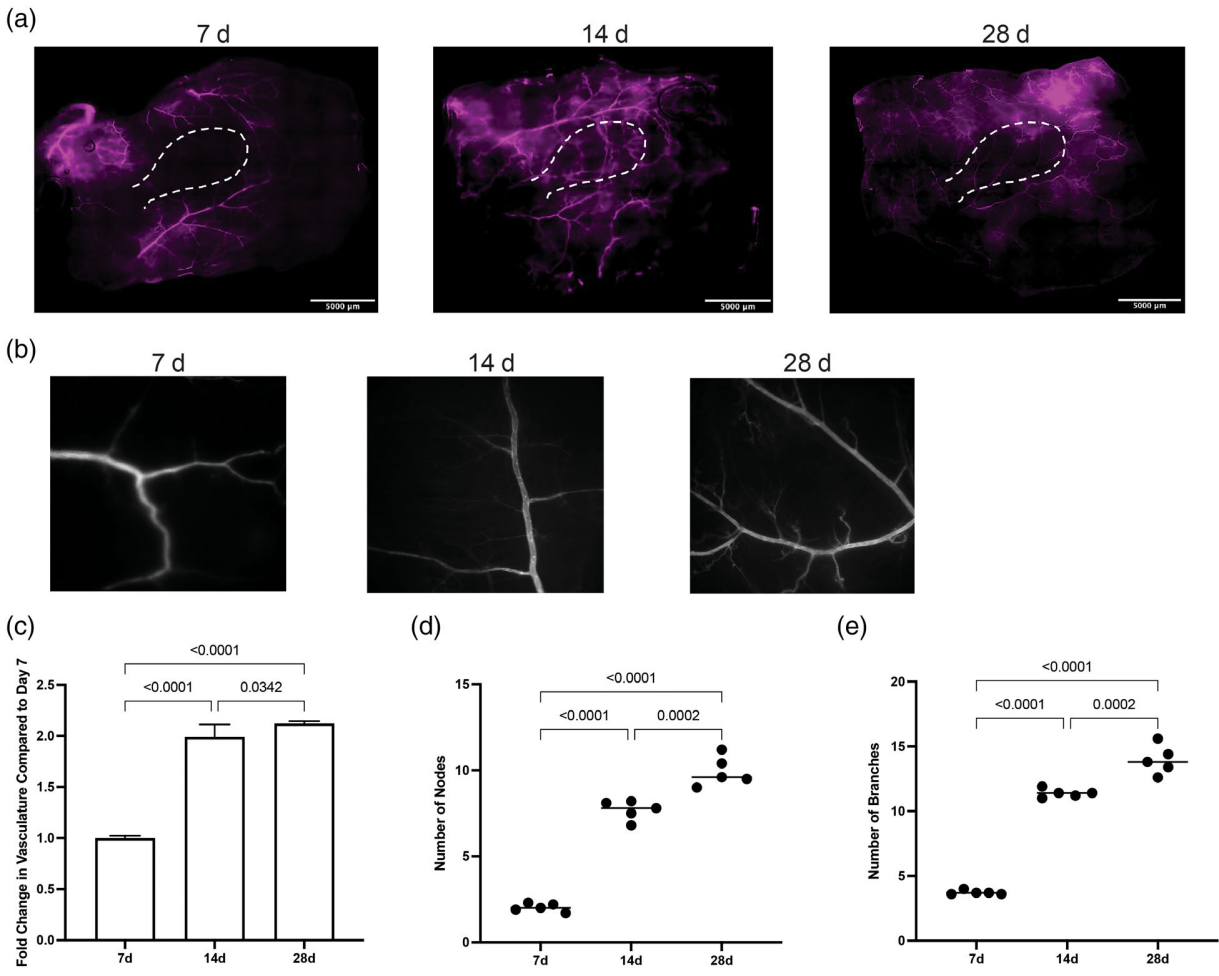
Vasculature formation around empty implanted devices. (a) Lectin‐perfusion assay (stained in purple) was performed to visualize functional vasculature after 7 (*n* = 6), 14 (*n* = 6), and 28 days (*n* = 6) of implantation. (b) Representative images of vascular networks (detected using lectin‐perfusion assay) used to quantify changes in (c) total vascular area, (d) number of nodes, and (e) number of branches around the implant. The significance across all experimental groups was performed using One‐way ANOVA, followed by Tukey's post hoc test.

Lectin perfusion assay was used to quantify vascular density around implanted empty devices at 1, 2, 4 weeks and compared the vascular density in the native skin of immunodeficient NSG mice (Figure 2a). At 1 week, no increase of vasculature was seen in the transplantation site. By 2 weeks, the total amount of functional vasculature around the implant doubled from baseline. After 2 weeks, the increase in vascular density diminishes, and only an additional ~10–15% increase in the percent vasculature is observed after 4 weeks of implantation (Figure 2a–c). The progression of angiogenesis was also evident by the increase in nodes and number of branches from pre‐existing blood vessels. After 2 weeks of implantation, nearly 3‐fold more nodes and branches were seen around the device compared with 1 week of implantation. Again, although there was an increase in the number of nodes and branches between Weeks 2 and 4, this increase was <1‐fold (Figure 2d,e). These results suggest that a vascularized site for islet engraftment can be created in the subcutaneous space after 2 weeks of implantation.

### 2.3 Survival of stem cell‐derived insulin‐producing beta cell clusters in prevascularized replenishable encapsulation devices

**FIGURE 3:**
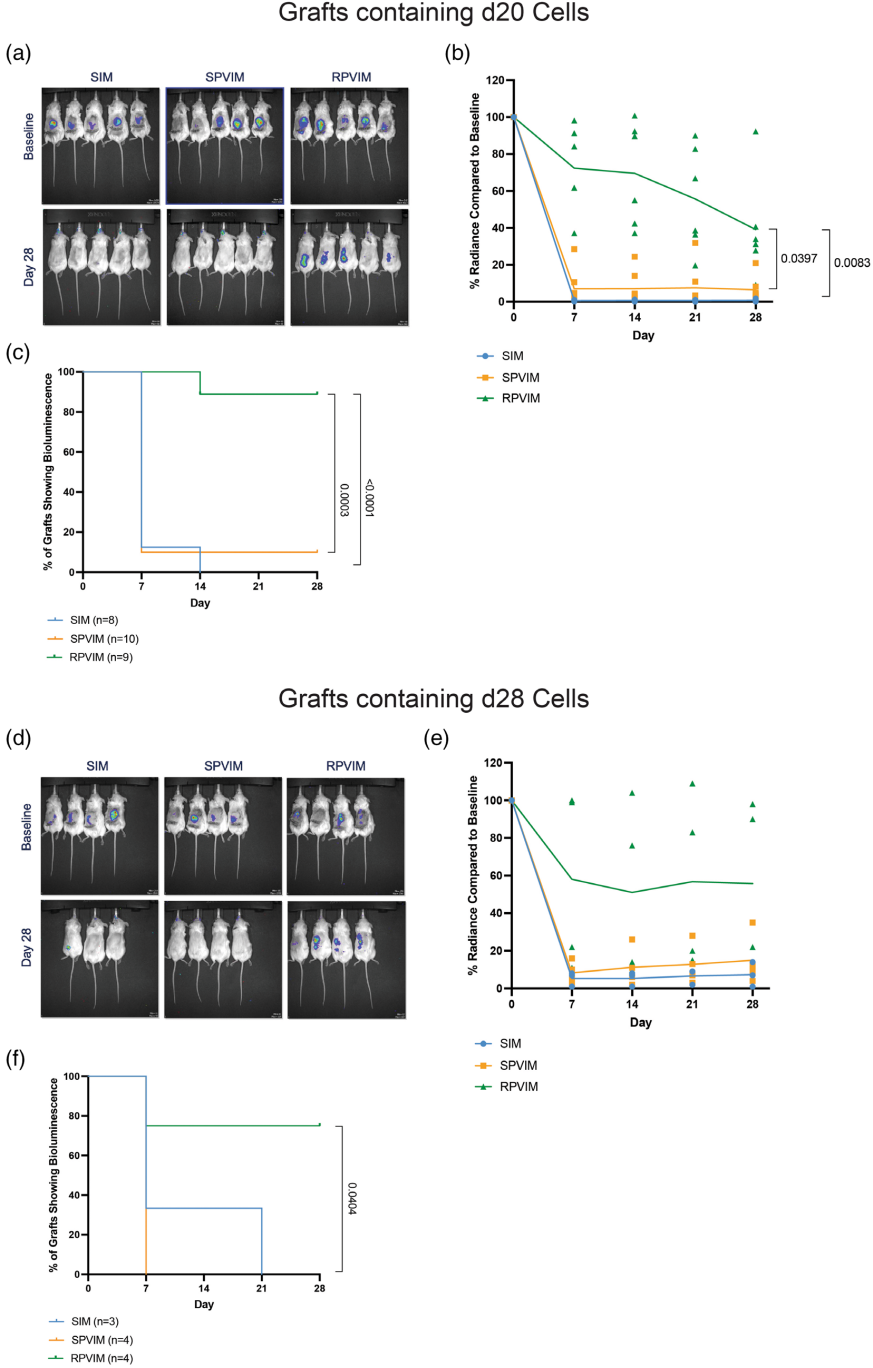
In vivo viability of stem cell‐derived insulin‐producing cells encapsulated in Refillable Prevascularized Implantation Method (RPVIM) devices. Representative images of encapsulated (a) Luciferase positive d20 cells in Standard Implantation Method (SIM) devices (*n* = 8, blue circles), Standard Prevascularization Method (SPVIM) devices (*n* = 10, orange squares), and RPVIM devices (*n* = 9, green triangles) and (d) Luciferase positive d28 in SIM devices (*n* = 3, blue circles), SPVIM devices (*n* = 4, orange squares), RPVIM devices (*n* = 4, green triangles). Quantification of bioluminescence signal from cells transplanted in devices compared with baseline for (B) d20 and (e) d28 cells. The significance of changes in bioluminescent signal at Day 28 versus baseline was determined using multiple unpaired *t*‐tests, corrected for multiple comparisons using Holm–Sidak method. Quantification of the percent of (c) d20 and (f) d28 grafts showing bioluminescence over a period of 28 days. The significance between survival curves was determined using the Kaplan–Meier test, and comparisons were made using a Log‐rank (Mantel–Cox) method.

We next asked whether prevascularization, with or without removal of the device, affects the survival of stem cell‐derived insulin‐producing beta cell clusters. These luciferase‐labeled cell clusters were generated using a previously established protocol.^47^, ^48^ For cell survival studies, two different stages of stem cell‐derived insulin‐producing beta cell clusters were used. Cells generated after 20 days of differentiation (d20) are immature beta cell‐like clusters, while Day 28 (d28) cells are mature beta cell‐like clusters that display enhanced functional properties (Figure S1).^47^, ^48^ Cells were encapsulated in devices and transplanted into the subcutaneous space of immunodeficient NSG mice, after which bioluminescence imaging was used to monitor the surviving graft mass of encapsulated cells over a period of 28 days. The d20 cells in SIM devices showed <15% of the baseline signal within the first 7 days, indicating that the initial transplantation site microenvironment was not hospitable for cells (Figure 3a). SPVIM devices also showed around 90% decrease in the average fractional change in bioluminescence signal compared with the baseline at Day 7. In comparison, the RPVIM devices showed only around 20% decrease in bioluminescence signal after Day 7, and these maintained >40% of the baseline bioluminescence signal after 28 days of implantation (Figure 3b). Additionally, when plotting the percentage of grafts showing >15% of the baseline bioluminescence signal across the different conditions, ~88% of the RPVIM devices showed a signal over 28 days (Figure 3c). This percentage was significantly greater than the SPVIM and SIM groups, indicating that the prevascularization without subsequent device removal improves survivability of immature stem cell‐derived insulin‐producing beta cell clusters in the subcutaneous space.

Moreover, this same trend was observed with the d28 cells, which are believed to be more susceptive to ischemia due to higher metabolic activity.^46^, ^47^ The change in bioluminescence compared with the baseline for cells in RPVIM was ~60% after 28 days, while the SIM and SPVIM showed only ~10%–15% fractional change (Figure 3d,e). The percent of grafts that survived by Day 28 for RPVIM was 75%; other conditions showed little to no survival after Day 7 (Figure 3f). These results support our hypothesis that prevascularization without vascular disruption creates a highly suitable posttransplant environment, allowing for increased graft survival.

### 2.4 Function of mature insulin‐producing cells within prevascularized replenishable encapsulation devices

**FIGURE 4:**
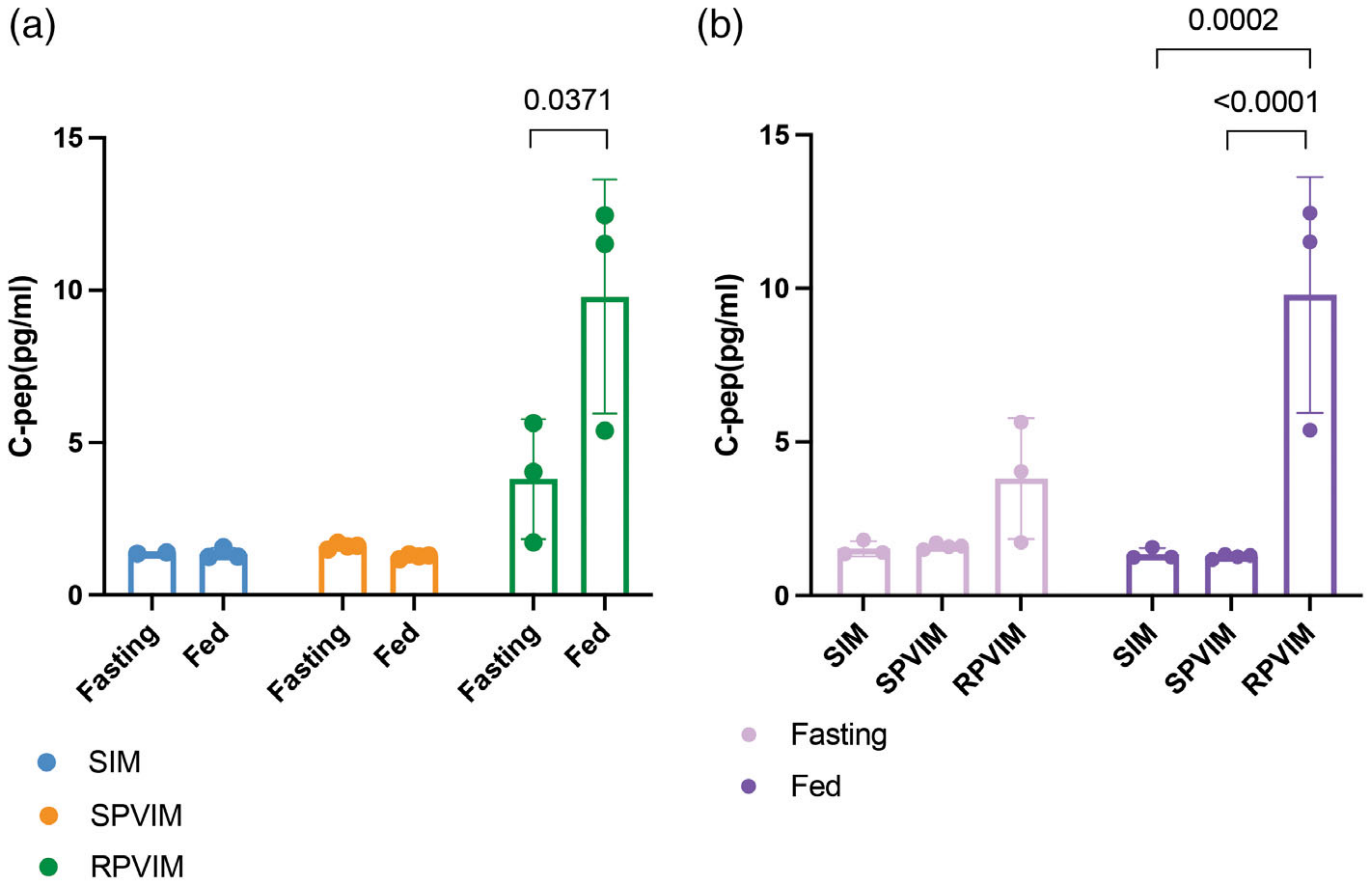
Glucose response and insulin secretion from RPVIM devices containing d28 stem cell‐derived insulin‐producing cells. (a) Levels of secreted C‐peptide from cells in Refillable Prevascularized Implantation Method (RPVIM) devices significantly increase 45 min postintraperitoneal glucose injection. The significance between fasting and glucose groups was determined using a one‐tailed unpaired *t*‐test. (b) Systemic C‐peptide levels in RPVIM devices are greater post‐IPGTT compared with Standard Implantation Method (SIM) and Standard Prevascularization Method (SPVIM) devices. Statistical significance across the groups was determined using a two‐way ANOVA fitting a mixed‐effects model followed by Tukey's post hoc test.

Given the viability data, we hypothesized that RPVIM devices would show greater glucose‐stimulated insulin secretion than SIM or SPVIM devices. Since the d20 cells are immature and do not respond significantly to glucose challenge, we conducted functional tests on d28 cells that more closely resemble mature beta‐cell‐like clusters and demonstrate glucose‐stimulate insulin secretion.^47^, ^48^ To assess the function of the encapsulated cells, after 28 days of implantation, we performed an intraperitoneal glucose tolerance test (IPGTT), in which mice were fasted overnight (“fasting”) and then challenged with a bolus intraperitoneal injection of 20 mM glucose (“fed”). d28 cells in the RPVIM device groups produced a significant increase of ~2‐fold more C‐peptide in the fed versus fasted state. In contrast, cells in the SIM and SPVIM groups showed no evidence of glucose‐stimulated insulin secretion (Figure 4a). Additionally, the amount of C‐peptide produced by cells in RPVIM devices postbolus of glucose was 5‐fold higher than cells in SIM and SPVIM groups. This postglucose response in RPVIM group was significantly greater than SIM and SPVIM groups (Figure 4b).

**FIGURE 5:**
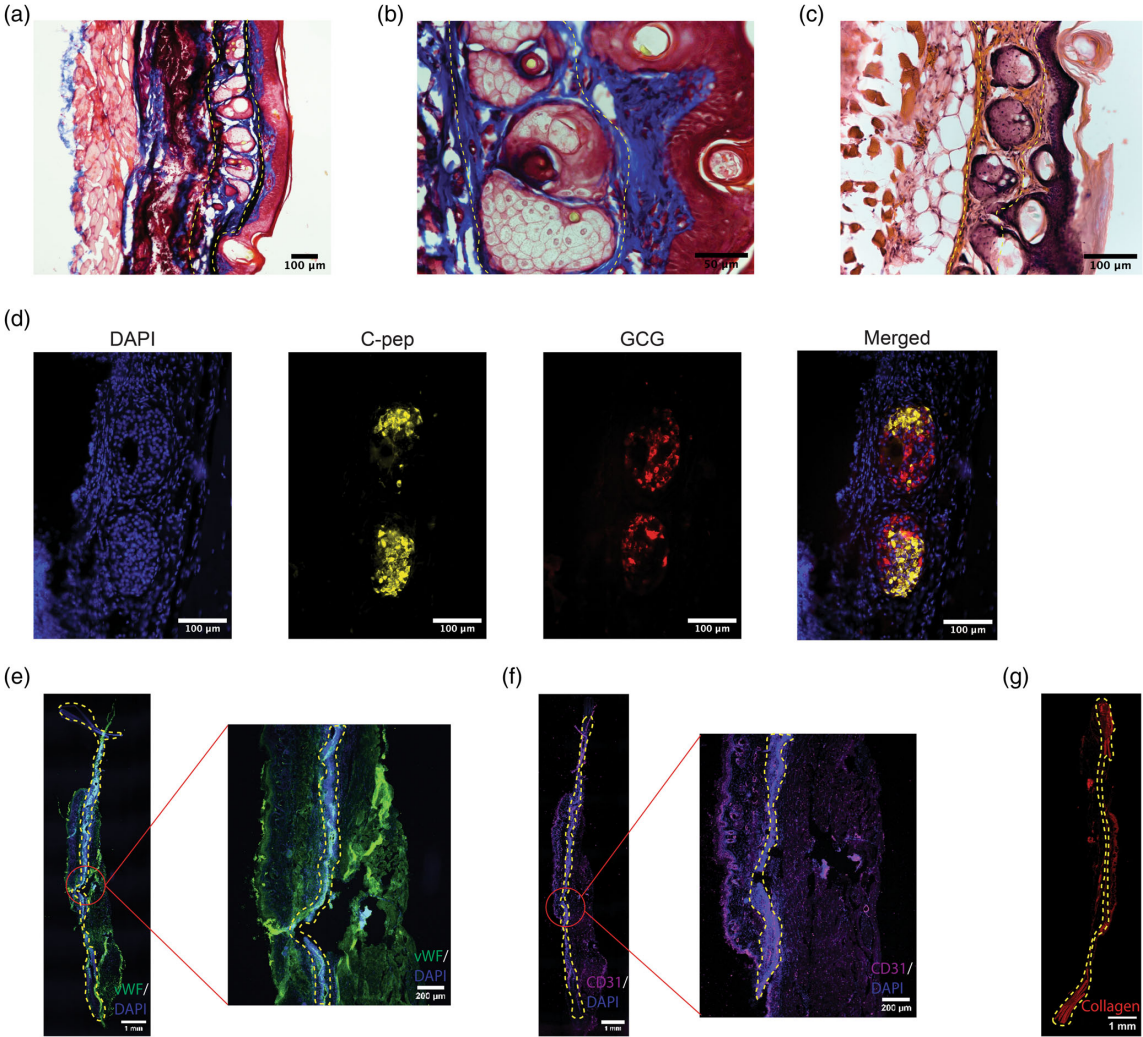
Histological analysis of Refillable Prevascularized Implantation Method (RPVIM) devices shows the presence of viable and functional d28 cells. In all the images, the outline of the device is shown using a yellow‐dashed line. (a) 20× and (b) 40× images of trichrome staining shows the presence of stem cell‐derived insulin‐producing beta cell clusters inside the device. The device resets between the skin and muscle layer, with numerous blood vessels surrounding the implant. (c) Representative 20× image of hematoxylin and eosin staining confirms the presence of islets and in vivo biocompatibility. (d) Representative immunofluorescence staining of stem cell‐derived insulin‐producing cells inside RPVIM device for human C‐peptide (C‐PEP, yellow), human glucagon (GCG, red), and nuclei (DAPI, blue). (e) Host vasculature (detected by staining with mouse‐specific anti‐von Willebrand Factor, green) is present around the outskirts of RPVIM device, showing the presence of neovasculature. Nuclei are stained with DAPI in blue. (f) Host endothelial cells (detected by mouse‐specific anti‐CD31 staining) are found primarily near the muscle layer in RPVIM devices. (g) Minimal collagen (detected by picrosirius red staining) is observed around the graft.

Explantation and histological analysis of RPVIM devices containing stem cell‐derived insulin‐producing beta cell clusters showed the presence of viable and insulin secreting beta‐like clusters inside the vascularized device. The trichrome staining showed multiple beta cell‐like clusters lined between the skin and muscle layers (Figure 5a,b). These beta cell‐like clusters are not found in the SIM and SPVIM device explants (Figures S2 and S3) The hematoxylin and eosin (H&E) staining show biocompatibility of the devices as there was no visible deposition of fibrotic tissue along the graft and no irregularities in size or shape at the tissue and cellular levels indicating that there are no detrimental effects caused by the implant (Figure 5c). Immunostaining of cells for glucagon and human C‐peptide confirmed the presence of islet‐like clusters within the device (Figure 5d). Additionally, like the H&E staining, picrosirius red staining also confirmed little to no levels of collagen present on the periphery of the device, confirming minimal fibrosis (Figure 5e). Immunofluorescence staining of von Willebrand Factor [vWF] and CD31 demonstrate increased angiogenesis and higher levels of platelet endothelial cell adhesion molecule 1, a marker for vascular differentiation (Figure 5f,g). Collectively, the IPGTT and histological analysis of grafts demonstrate robust glucose‐stimulated insulin secretion and morphological integrity of stem cell‐derived insulin‐producing beta cell clusters within the RPVIM devices.

## 3 DISCUSSION

Our results confirm other studies reporting that implantation within the subcutaneous space does not allow for the survival of beta cells, which are typically highly vascularized in their native environment.^5^, ^19^, ^40^, ^48^, ^49^ Low oxygen tension and lack of nutrient supply caused by insufficient vasculature often lead to ischemia and necrosis of the highly metabolic beta cells.^2^, ^4^, ^24^, ^25^, ^39^, ^50^, ^51^ It has also been shown that beta cell function and insulin secretion are severely impacted by the dense vascular network through blood flow‐dependent and independent pathways.^5^, ^38^, ^49^ Therefore, it is crucial for encapsulated beta cells to have functional and robust vasculature around the implant.

Here, we demonstrate an easy‐to‐implement prevascularization approach that aims to relieve the ischemic stress experienced by encapsulated cells upon implantation in immunodeficient NSG mice. Our results indicate that in the subcutaneous space, prevascularization of replenishable devices increases the survival of encapsulated stem cell‐derived insulin‐producing beta cell clusters posttransplantation. The nanoporous thin‐film encapsulation device was designed such that after prevascularization, cells could be loaded easily through the port without the need to remove the device from the transplant site. After the cells were loaded, the device was resealed using resistive heating. This technique maintains the device's shape and structure while also preserving the surrounding vasculature (Figure 1). Additionally, previously published research suggested that a functional, planar encapsulation device should be no more than 550 μm in thickness with a volume fraction of ~2.5%.^52^ In our encapsulation devices, the total volume of transplanted beta cell cluster mass was 4.423 μL, which results in a volume fraction of ~2.7%. Also, we estimated that these loaded encapsulation devices had a thickness of ~100 μm, demonstrating that our device design satisfies the optimized parameters. Analysis of the vasculature around the encapsulation device showed that after 14 days of prevascularization in the subcutaneous space, the device is surrounded with twice as many new, functional blood vessels compared with 7 days of implantation (Figure 2). The presence of these blood vessels plays an important role in cell survival and function inside the encapsulation device, as we observed greater than 80% graft survival in RPVIM devices in the subcutaneous space (Figure 3). The RPVIM devices also show greater performance than SPVIM devices, a technique used previously by several groups to enhance cell engraftment.^7^, ^20^, ^32^, ^39^, ^42^ This was also observed in more mature stem cell‐derived insulin‐producing beta cell clusters, which are highly metabolic and require robust sources of nutrients and oxygen.^46^, ^47^ Moreover, the mature cells in prevascularized grafts also demonstrate significantly higher levels of glucose‐stimulated insulin secretion compared with controls (Figure 4). The histological analysis demonstrates in vivo biocompatibility and the presence of functional stem cell‐derived insulin‐producing beta cell clusters inside RPVIM devices (Figure 5). While functional beta cell clusters were observed in the device, some necrotic areas were also present, likely due to insufficient oxygen supply. This is also indicated by the 50% reduction in cluster mass observed in RPVIM devices (Figure 3). However, overall, the RPVIM approach resulted in a significant increase in vascular coverage and oxygen availability, leading to better preservation of beta cell cluster mass and function compared with controls. Results from this study confirm that the RPVIM devices effectively provide a hospitable microenvironment for encapsulated stem cell‐derived insulin‐producing beta cell clusters in the subcutaneous space for 28 days. Future studies include longer‐term survival and function studies using the RPVIM devices and testing the ability of these devices to reverse diabetes in a diabetic mouse model. These studies will provide key insights into the clinical benefit of this approach.

Our approach involves modification of the subcutaneous site to create a highly vascularized implantation site without introducing multiple devices, materials, or biologics whose long‐term safety and biocompatibility need to be accounted for. This strategy utilized only FDA‐approved biomaterials and took advantage of the naturally occurring vascularization process in the body.^24^, ^44^, ^45^ The lectin perfusion studies demonstrated functional vasculature surrounding the implant at 14 days, which we deemed sufficient time to create a favorable transplantation site. We observed robust vascular response with vessel branching, ingrowth, and outgrowth on and around the encapsulation device in the subcutaneous site. Additionally, since neovascularization is a dynamic process, we expect the vasculature to remodel extensively over time, especially with the addition of encapsulated cells.^5^, ^49^ Furthermore, our approach showed that not only is a prevascularized site necessary for subcutaneous implants, but it is also crucial to ensure that we preserve the vascular network while transplanting the cells in the prevascularized site.

## 4 CONCLUSION

Islet transplantation using cell encapsulation devices can offer a long‐term treatment for T1D. A major limitation that remains unaddressed is the loss of cell viability posttransplant. A major cause of this is the ischemic stress experienced by encapsulated cells that are transplanted in the inherently low vascularized subcutaneous space. However, transplantation of cellular grafts in the subcutaneous space is highly attractive as it allows for noninvasive graft monitoring, ease in device retrieval, or re‐filling the implanted device with new islets if required. Previous research has shown that modifying the subcutaneous space to increase vascular network formation prior to the transplantation of encapsulation devices can increase graft survival and function. However, these approaches often involve the use of biologics or techniques that disrupt the vasculature when inserting the encapsulation device.

Our replenishable prevascularized device allows the transplantation of insulin‐producing cells in encapsulation devices without disrupting the hospitable environment formed around the device during the prevascularization period. Thus, we can take advantage of the body's ability to form robust vasculature around PCL devices. Our results show that this method increases the survival and function of cells encapsulated within thin‐film PCL devices in the subcutaneous space over 28 days. We anticipate that the results of this work are relevant to a broader range of cell therapy devices in which ischemic stress leads to a loss in graft posttransplantation.

## 5 MATERIALS AND METHODS

### 5.1 Device fabrication

Chemicals were purchased from Sigma‐Aldrich unless otherwise noted. Porous PCL thin films were fabricated by dissolving PCL (80 kDa Mn) and poly (ethylene) glycol (2 kDa Mn) in 2,2,2‐trifluoroethanol. The 150 mg/mL PEG:PCL solution was spun and cast onto silicon wafers for 90 s at 1000 RPM, after which the films were immersed in water to allow for PEG leaching and pore formation. To fabricate the device, two ~10 μm thick, porous, thin films were sandwiched onto a polydimethylsiloxane (PDMS) Sylgard 40 mold with a spoon‐shaped nichrome wire embedded. The two films fused through resistive heating by passing a 1.2 A current through the nichrome wire, creating a spoon‐shaped device with an opening at the neck. The resulting device was 1.5 cm in diameter, with a final 1 cm long neck that resulted in a total volume capacity of 160 μL.

A thicker PCL film (spun cast on silicon wafers for 30 s at 300 RPM) was used to create a wider border around the device. For devices that were prevascularized, a small cannula was placed inside the device and sealed. For devices that were not prevascularized, the opening was sealed using resistive heating after cells in 70 μL of cell medium were pipetted inside the device. Although the total volume capacity was 160 μL, we only added 70 μL to ensure proper sealing and no spillage of cells when cells were inserted in RPVIM devices in vivo.

### 5.2 Mice

NOD.Cg‐*Prkdc* ^*scid*^ *Il2rg* ^*tm1Wjl*^/SzJ mice (NSG; Jackson Strain 005557) mice were purchased from Jackson Laboratories. Mice used in this study were housed and handled according to ethical guidelines approved by the Institutional Animal Care and Use Committee (IACUC) at the University of California, San Francisco Laboratory Animal Resource Center (LARC).

### 5.3 Culture of pluripotent cells

A previously published gene‐targeting approach of the insulated human AAVS1 loci was used to generate a cell line that expresses a constitutive firefly luciferase gene. Cell culture was performed using the NIH‐approved human embryonic stem cell (hESC) line MEL‐1 (NIH registration number: 0139), having GFP knocked into one allele of the endogenous insulin locus (INS^GFP/W^ hESCs; Micallef et al., 2011). INS^GFP/W^ hESCs were cultured on irradiated mouse embryonic fibroblasts (Thermo Fisher) in hESC maintenance media composed of Dulbecco's Modified Eagle Medium (DMEM)/F12, 20% (vol/vol) KnockOut serum replacement (Thermo Fisher Scientific), nonessential amino acids (Thermo Fisher Scientific), GlutaMAX (Thermo Fisher Scientific), and 2‐mercaptoethanol (Millipore). The maintenance media was supplemented with 4 ng/mL recombinant human FGF‐2 (R&D Systems). Confluent hESCs were dissociated into single‐cell suspension by incubation with TrypLE Select (Gibco) and passaged every 3–4 days. G‐banded karyotyping performed by Cell Line Genetics confirmed normal karyotype of INS^GFP/W^ hESCs. Cells have been confirmed to be mycoplasma‐free using the MycoProbe Mycoplasma Detection Kit (R&D Systems) or the Venor GeM Mycoplasma Detection Kit (Sigma).

### 5.4 Differentiation into pancreatic cells

To initiate differentiation, we dissociated confluent cultures into single‐cell suspensions using TrypLE Select, counted them, and seeded them in six‐well suspension plates at a density of 5.5 × 10^6^ cells per 5.5 mL of hESC maintenance media supplemented with 10 ng/mL activin A (R&D Systems) and 10 ng/mL heregulinB (PeproTech). The plates were incubated at 37°C and 5% CO_2_ on an orbital shaker set at 100 rpm to induce 3D sphere formation. After 24 h, the spheres were differentiated as previously described.^47^ Spheres were collected in 50‐mL tubes, allowed to settle by gravity, washed once with PBS or Roswell Park Memorial Institute (RPMI) medium (Gibco), and resuspended in d1 differentiation media. The resuspended spheres were distributed into fresh six‐well suspension plates for a final volume of 5.5 mL of d1 media per well. Until d3, spheres were fed daily by removing media and replenishing with 5.5 mL of fresh media. From d4 to d20, media was removed daily, and 5 mL of fresh media was added. Media compositions for differentiation of INS^GFP/W^ hESCs are as follows: d1, RPMI (Gibco) containing 0.2% fetal bovine serum (FBS), 1:5000 insulin transferrin selenium G (ITS‐G, Gibco), 100 ng/mL activin A, and 50 ng/mL WNT3a (R&D Systems); d2, RPMI containing 0.2% FBS, 1:2000 ITS, and 100 ng/mL activin A; d3, RPMI containing 0.2% FBS, 1:1000 ITS, 2.5 μM TGFbi IV (Calbiochem), and 25 ng/mL keratinocyte growth factor (KGF; R&D Systems); d4–5, RPMI containing 0.4% FBS, 1:1000 ITS, and 25 ng/mL KGF; d6–7, DMEM (Gibco) with 25 mM glucose containing 1:100 B27 (Gibco) and 3 nM TTNPB (Sigma); d8, DMEM with 25 mM glucose containing 1:100 B27, 3 nM TTNPB, and 50 ng/mL epidermal growth factor (EGF; R&D Systems); d9–11, DMEM with 25 mM glucose containing 1:100 B27, 50 ng/mL EGF, and 50 ng/mL KGF; d12+, DMEM with 25 mM glucose containing 1:100 B27, 1:100 GlutaMAX (Gibco), 1:100 NEAA (Gibco), 10 μm ALKi II (Axxora), 500 nM LDN‐193189 (Stemgent), 1 μm Xxi (Millipore), 1 μM T3 (Sigma‐Aldrich), 0.5 mM vitamin C, 1 mM *N*‐acetylcysteine (Sigma‐Aldrich), 10 μM zinc sulfate (Sigma‐Aldrich), and 10 μg/mL heparin sulfate.

For transplantation experiments for in vivo imaging system (IVIS) imaging, MEL1 INS^GFP/W^ hESCs differentiated up to d19–20, while for in vivo glucose measurement experiments, the cells were cultured for an additional 7 days in Connaught Medical Research Laboratories (CMRL) medium containing 10% FBS, 1:100 Glutamax (Gibco), 1:100 NEAA (Gibco), 10 μm ALKi II (Axxora), 0.5 mM vitamin C, 1 μM T3 (Sigma‐Aldrich), 1 mM N‐acetyl Cysteine (Sigma‐Aldrich), 10 μM zinc sulfate (Sigma‐Aldrich), and 10 μg/ml of heparin sulfate supplemented with 10 μM of the ROCK inhibitor Y‐27632 (Tocris) and Penicillin Streptomycin (Corning) before transplantation.

### 5.5 Transplantation

PCL encapsulation devices were implanted in the subcutaneous space via a small incision in the mouse's lower back. The space between the skin and the muscle layer was dissected using blunt forceps, and saline was injected to create an easily accessible pocket. The encapsulation device was implanted in this newly formed space, and the skin wound was closed using surgical staples. For devices containing cells, 2.5 × 10^3^ clusters in 70 μL of media were transplanted. For prevascularized devices, a small incision was made near the previous incision site, and a blunt instrument was used to open the space such that the neck of the device was visible. Without disrupting the device's position, scissors were used to cut open the neck, and a 200 μL pipet tip was used to insert cells, after which the cannula inside the device was removed, and a cautery pen was used to seal the device.

### 5.6 Lectin perfusion to assess functional vasculature

To analyze the vasculature around the grafts, lectin perfusion was performed on mice containing empty (no cell) devices. 0.1 mL of 1 mg/mL Tomato‐lectin Dylight 647 (Thermo Fisher) was injected into the atrium of mice after anesthetizing with isoflurane. The right atrium of the mouse was cut, and after the mouse was allowed to bleed out, 10 mL of PBS was injected into the left ventricle using a pump. Finally, 10 mL of 4% paraformaldehyde (PFA) was injected into the left ventricle using the pump, after which the device and surrounding skin were dissected and stored in 4% PFA overnight. Samples were transferred to 30% sucrose, washed with PBS, and imaged using a Nikon 6D/High‐throughput microscope and a Leica Widefield microscope. To quantify the vasculature around the entire device, 4× magnification images were used. These acquired images were analyzed using the Fiji software (NIH, version 2.0.0‐rc‐69/1.53c). Specifically, the Fiji software set a manual threshold for each image, after which the area covered by the stained vessels was manually highlighted and then calculated by the program. This area was divided by the total area of the image to determine the percentage of vasculature over the total area. To determine the fold change in the vasculature, all values were divided by the average percentage of vasculature over the total area on Day 7. For each time point, *n* = 5 animals were used, and five images of 4× magnifications per animal were used for the analysis. The number of branches and nodes was manually calculated for each image. A node was defined as a point at which two vessels intersect, and a branch was defined as vessels that were extending from the main blood vessel.

### 5.7 Bioluminescent imaging

Graft‐bearing animals were injected IP with D‐luciferin solution (Goldbio) at 150 mg/kg 30 min before imaging to capture the peak in bioluminescent intensity. Mice were anesthetized with an isoflurane mixture (2% in 98% O_2_), and the bioluminescent signal was quantified using a Xenogen IVIS 200 imaging system (PerkinElmer). Images were acquired for 1 min and then analyzed using the Living Image analysis software (Xenogen). Regions of interest (ROIs) were centered over the location of the devices, and background signal was obtained by capturing the ROI of a nonbioluminescent signal. Photons collected over the acquisition time were counted within the ROI. The same imaging protocol was applied each time to ensure consistency across longitudinal studies.

### 5.8 Intraperitoneal glucose tolerance test

Mice were subjected to an IPGTT at 28 days posttransplantation to assess function of the grafted cells. The test was split into two parts to prevent undue stress on mice. In the first round, mice were fasted overnight, after which the blood glucose levels and blood samples were obtained via tail vein and cheek bleeds, respectively. After 4 days of rest, mice were fasted overnight, and blood glucose levels were measured from the tail vein. Three milligrams per kilogram of glucose was injected into the intraperitoneal space of mice, and after 45 min, blood glucose and blood samples were obtained from the tail vein, and cheek bleeds, respectively. Cpeptide levels were measured using an ultrasensitive insulin ELISA kit (Alpco 80‐CPTHU‐CH05).

### 5.9 Histology

Explanted grafts were collected and fixed in 4% PFA for 24 h and dehydrated in 30% sucrose for 48 h. Tissue samples were embedded in optimal cutting temperature (OCT) and 8 μm sections were placed on TOMO adhesive slides for immunostaining. Slides were stained with H&E (StatLab), picrosirius red (Polysciences), and tri‐chrome (Sigma) staining kits. Images for H&E and trichrome staining were obtained on a brightfield microscope, and picrosirius red staining was visualized using a circular polarized light microscope. For immunostaining, slides were fixed in 95% methanol and permeabilized in 0.1% Triton X‐100. Sections were treated with primary antibodies against CD31 (R&D systems AF3628) diluted 1:50, vWF (Millipore AB7356) diluted 1:10, and C‐peptide (DSHB GN‐ID4) diluted 1:200, overnight at 4 C. Samples were rinsed with PBS followed by 1 h room temperature incubation with 1:500 dilution of secondary antibody (AF 546, Sigma) and/or Alexa Fluor 647‐conjugated glucagon antibody (Novus Biologics IC1249R) diluted 1:2000. Samples were then rinsed with PBS, incubated with Hoechst 3322 for 5 min, and mounted using anti‐fade mounting medium (ProLong, LifeTechnologies). Images were obtained using a NIKON widefield fluorescence microscope.

### 5.10 Statistical analysis

Data were analyzed using GraphPad Prism software version 9.4. All differences in vasculature between experimental groups were evaluated using one‐way analysis of variance (ANOVA) or Two‐way ANOVA, followed by Tukey's post hoc test or Student's *t*‐test. Graft survival was compared using Kaplan–Meier survival curves. A *p* < 0.05 was considered statistically significant.

## AUTHOR CONTRIBUTIONS

**Gauree Chendke:** Conceptualization (equal); data curation (equal); formal analysis (lead); methodology (equal); writing – original draft (lead); writing – review and editing (lead). **Bhushan Kharbikar:** Conceptualization (equal); data curation (equal); methodology (equal); project administration (equal); writing – review and editing (supporting). **Sudipta Ashe:** Data curation (supporting); formal analysis (supporting); writing – review and editing (supporting). **Gaetano Faleo:** Conceptualization (equal); data curation (supporting); methodology (supporting); writing – review and editing (supporting). **Julie B. Sneddon:** Conceptualization (supporting); funding acquisition (supporting); project administration (supporting); resources (supporting); writing – review and editing (supporting). **Qizhi Tang:** Conceptualization (supporting); project administration (supporting); resources (supporting); writing – review and editing (supporting). **Matthias Hebrok:** Funding acquisition (equal); resources (equal); supervision (equal); visualization (equal); writing – review and editing (supporting). **Tejal Desai:** Conceptualization (equal); funding acquisition (equal); project administration (lead); resources (equal); writing – review and editing (equal).

## CONFLICT OF INTEREST STATEMENT

Tejal Desai is a scientific founder of Encellin, a cell therapy device company, and she is listed as an inventor of a thin film macro‐encapsulation technology (US Patent no. 10,865,378). Julie B. Sneddon has served on the Scientific Advisory Board (SAB) of Encellin Inc. No direct funds have been given to Julie B. Sneddon or her lab by Encellin Inc. Qizhi Tang is a SAB member and holds stocks in Encellin Inc and Minutia Inc. Matthias Hebrok holds stocks in Encellin Inc, Thymmune Therapeutics Inc, and has received research support from Eli Lilly. He is the co‐founder and SAB member of Minutia Inc. and holds stocks and options in the company.

## Supporting information


**FIGURE S1:** Schematic showing differentiation of stem cell‐derived insulin‐producing beta cell clusters. The beta cell clusters are derived from human embryonic pluripotent stem cells and are differentiated to produce immature beta cell‐like clusters (d20) or mature beta cell‐like clusters (d28).
**FIGURE S2:** Histological analysis of Standard Implantation Method (SIM) devices shows lack of stem cell‐derived insulin‐producing beta cell clusters. In all the images, the outline of the device is shown using a yellow‐dashed line. (a) 20× images of trichrome staining shows that there are no beta cell clusters as seen in SIM devices. (b) 4× and representative 2× image of hematoxylin and eosin staining confirms the in vivo biocompatibility of the SIM devices. (c) Representative immunofluorescence staining of stem cell‐derived insulin‐producing cells inside Refillable Prevascularized Implantation Method device for human C‐peptide (C‐PEP, yellow), human glucagon (GCG, red), and nuclei (DAPI, blue). No signal for human C‐PEP and/or human glucagon was seen. (d) Negligible host vasculature (detected by staining with mouse‐specific anti‐von Willebrand Factor [vWF], green) is present around the outskirts of SIM devices. Nuclei are stained with DAPI in blue. (e) Host endothelial cells (detected by mouse specific anti‐CD31 staining) are found primarily near the muscle layer in SIM devices.
**FIGURE S3:** Histological analysis of Standard Prevascularization Method (SPVIM) devices shows similar results as Standard Implantation Method (SIM) devices. In all the images, the outline of the device is shown using a yellow‐dashed line. (a) 20× image of trichrome staining shows that there are no beta cell clusters as seen in SPVIM devices. (b) 4× and representative 2× image of hematoxylin and eosin staining confirms the in vivo biocompatibility of the SPVIM devices. (c) Representative immunofluorescence staining of stem cell‐derived insulin‐producing cells inside SPVIM device for human C‐peptide (C‐PEP, yellow), human glucagon (GCG, red), and nuclei (DAPI, blue). No signal for human C‐peptide and/or human glucagon was seen. (d) Little to no amount of host vasculature (detected by staining with mouse‐specific anti‐von Willebrand Factor [vWF], green) is present around the outskirts of SIM devices. Nuclei are stained with DAPI in blue. (e) Host endothelial cells (detected by mouse specific anti‐CD31 staining) are found primarily near the muscle layer in Refillable Prevascularized Implantation Method devices.

## Data Availability

The data that support the findings of this study are available from the corresponding author upon reasonable request.
