# Supplementary material for: Replenishable prevascularized cell encapsulation devices increase graft survival and function in the subcutaneous space
Source: Bioeng Transl Med. 2023 May 19;8(4):e10520. doi: 10.1002/btm2.10520 (PMC10354771; doi:10.1002/btm2.10520)
Supplement: Supplementary file 1 — FIGURE S1: Schematic showing differentiation of stem cell‐derived insulin‐producing beta cell clusters. The beta cell clusters are derived from human embryonic pluripotent stem cells and are differentiated to produce immature beta cell‐like clusters (d20) or mature beta cell‐like clusters (d28). FIGURE S2: Histological analysis of Standard Implantation Method (SIM) devices shows lack of stem cell‐derived insulin‐producing beta cell clusters. In all the images, the outline of the device is shown using a yellow‐dashed line. (a) 20× images of trichrome staining shows that there are no beta cell clusters as seen in SIM devices. (b) 4× and representative 2× image of hematoxylin and eosin staining confirms the in vivo biocompatibility of the SIM devices. (c) Representative immunofluorescence staining of stem cell‐derived insulin‐producing cells inside Refillable Prevascularized Implantation Method device for human C‐peptide (C‐PEP, yellow), human glucagon (GCG, red), and nuclei (DAPI, blue). No signal for human C‐PEP and/or human glucagon was seen. (d) Negligible host vasculature (detected by staining with mouse‐specific anti‐von Willebrand Factor [vWF], green) is present around the outskirts of SIM devices. Nuclei are stained with DAPI in blue. (e) Host endothelial cells (detected by mouse specific anti‐CD31 staining) are found primarily near the muscle layer in SIM devices. FIGURE S3: Histological analysis of Standard Prevascularization Method (SPVIM) devices shows similar results as Standard Implantation Method (SIM) devices. In all the images, the outline of the device is shown using a yellow‐dashed line. (a) 20× image of trichrome staining shows that there are no beta cell clusters as seen in SPVIM devices. (b) 4× and representative 2× image of hematoxylin and eosin staining confirms the in vivo biocompatibility of the SPVIM devices. (c) Representative immunofluorescence staining of stem cell‐derived insulin‐producing cells inside SPVIM device for human C‐peptide (C‐ [file BTM2-8-e10520-s001.docx]

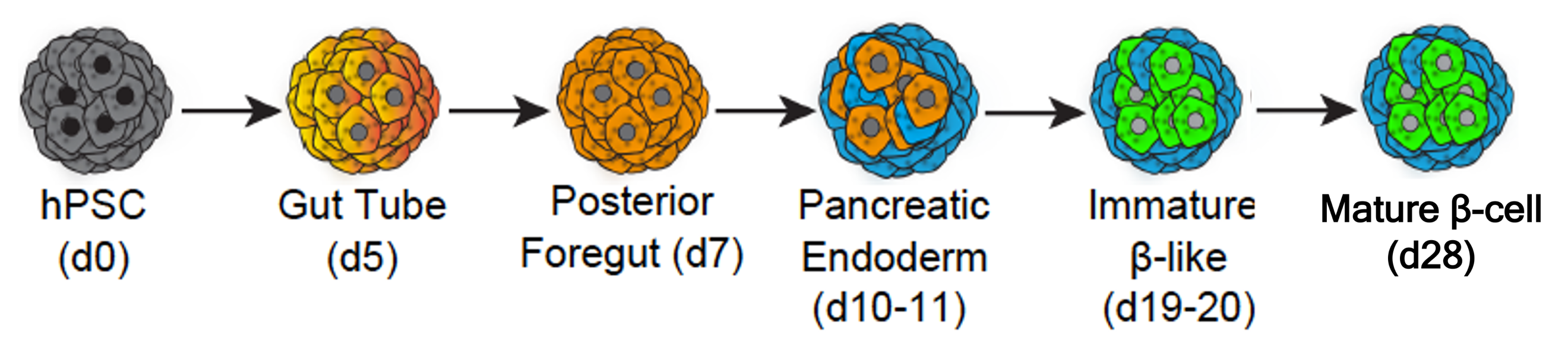


**Figure S1.** Schematic showing differentiation of stem cell-derived insulin-producing beta cell clusters. The beta cell clusters are derived from human embryonic pluripotent stem cells and are differentiated to produce immature beta cell-like clusters (d20) or mature beta cell-like clusters (d28).


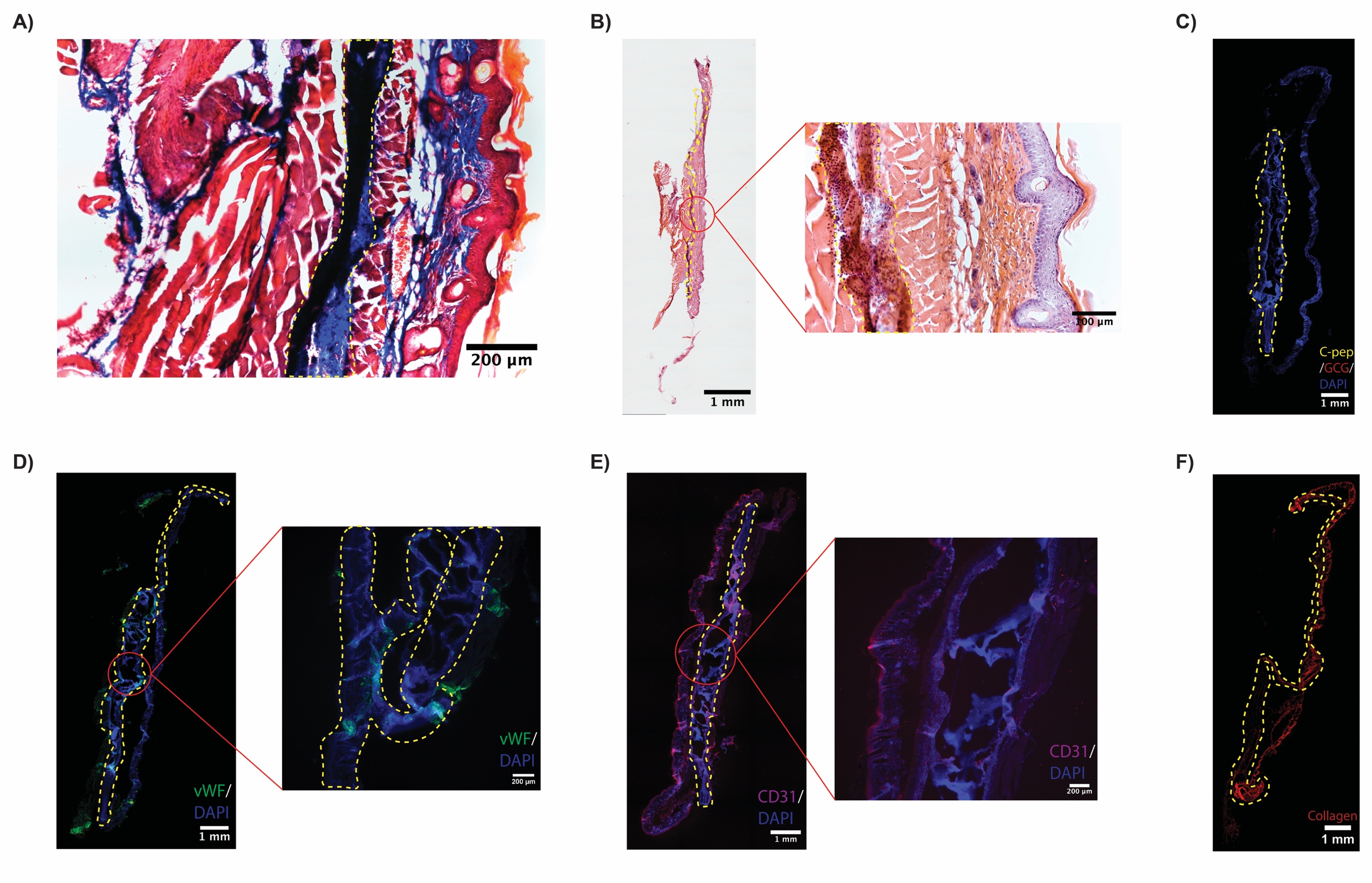


**Figure S2.** Histological analysis of SIM devices shows lack of stem cell-derived insulin-producing beta cell clusters. In all the images, the outline of the device is shown using a yellow dashed line. A) 20x images of trichrome staining shows that there are no beta cell clusters as seen in SIM devices. B) 4X and representative 2X image of H&E staining confirms the *in vivo* biocompatibility of the SIM devices. C) Representative immunofluorescence staining of stem cell-derived insulin-producing cells inside RPVIM device for human C-peptide (C-PEP, yellow), human glucagon (GCG, red), and nuclei (DAPI, blue). No signal for human C-peptide and/or human glucagon was seen. D) Negligible host vasculature (detected by staining with mouse-specific anti-vWF, green) is present around the outskirts of SIM devices. Nuclei are stained with DAPI in blue. E) Host endothelial cells (detected by mouse specific anti-CD31 staining) are found primarily near the muscle layer in SIM devices.


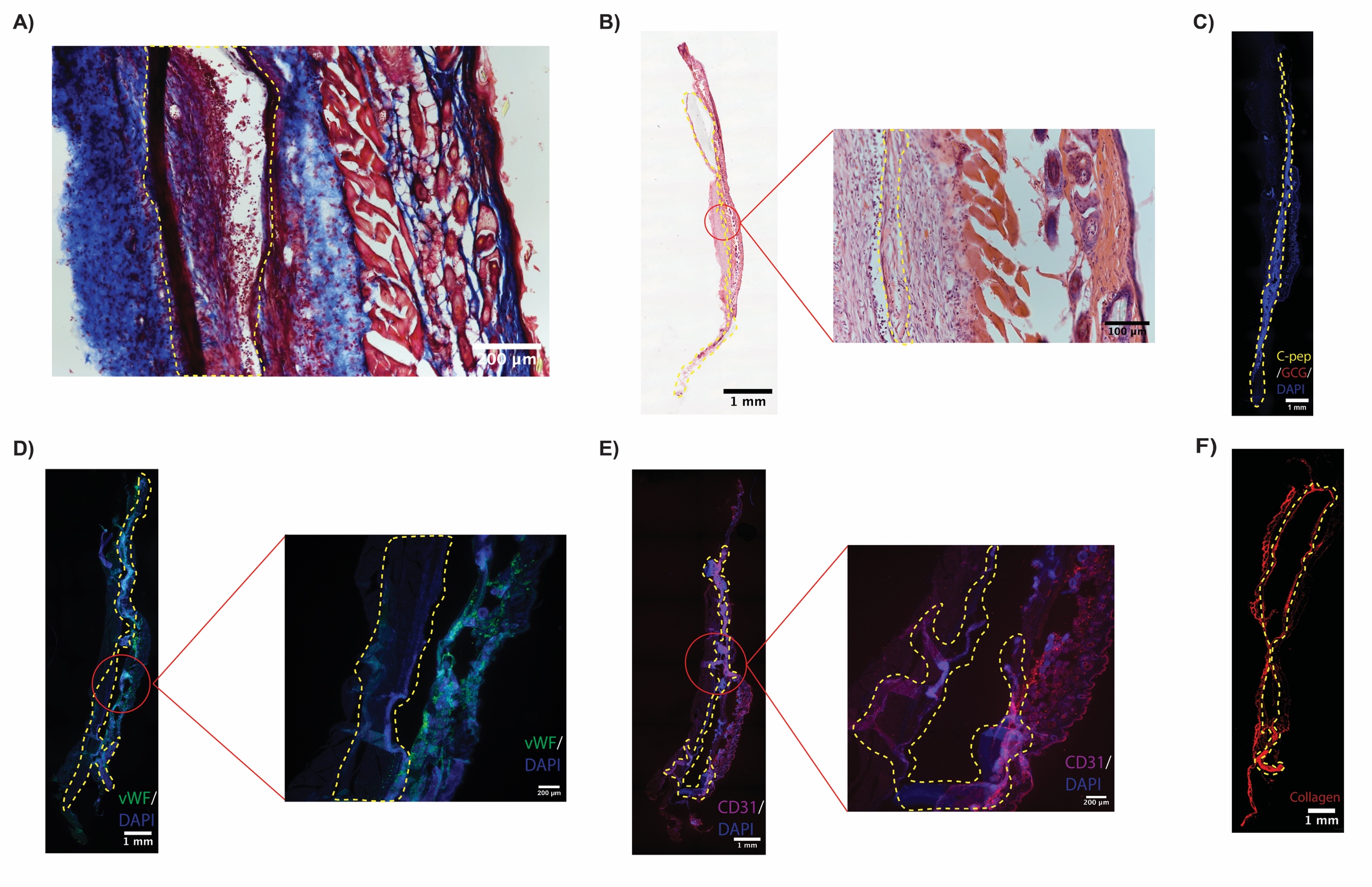


**Figure S3.**  Histological analysis of SPVIM devices shows similar results as SIM devices. In all the images, the outline of the device is shown using a yellow dashed line. A) 20x image of trichrome staining shows that there are no beta cell clusters as seen in SPVIM devices. B) 4X and representative 2X image of H&E staining confirms the *in vivo* biocompatibility of the SPVIM devices. C) Representative immunofluorescence staining of stem cell-derived insulin-producing cells inside SPVIM device for human C-peptide (C-PEP, yellow), human glucagon (GCG, red), and nuclei (DAPI, blue). No signal for human C-peptide and/or human glucagon was seen. D) Little to no amount of host vasculature (detected by staining with mouse-specific anti-vWF, green) is present around the outskirts of SIM devices. Nuclei are stained with DAPI in blue. E) Host endothelial cells (detected by mouse specific anti-CD31 staining) are found primarily near the muscle layer in RPVIM devices.
